# MicroRNA-1 Downregulation Increases Connexin 43 Displacement and Induces Ventricular Tachyarrhythmias in Rodent Hypertrophic Hearts

**DOI:** 10.1371/journal.pone.0070158

**Published:** 2013-07-26

**Authors:** Antonio Curcio, Daniele Torella, Claudio Iaconetti, Eugenia Pasceri, Jolanda Sabatino, Sabato Sorrentino, Salvatore Giampà, Mariella Micieli, Alberto Polimeni, Beverley J. Henning, Angelo Leone, Daniele Catalucci, Georgina M. Ellison, Gianluigi Condorelli, Ciro Indolfi

**Affiliations:** 1 Division of Cardiology, Laboratory of Molecular and Cellular Cardiology, Department of Medical and Surgical Sciences, University Magna Graecia, Catanzaro, Italy; 2 URT - National Research Council, University Magna Graecia, Catanzaro, Italy; 3 Biostem Unit, RISES, Liverpool John Moores University, Liverpool, United Kingdom; 4 Humanitas Clinical and Research Center, Rozzano, Milan, and National Research Council, Italy; Goethe University, Germany

## Abstract

Downregulation of the muscle-specific microRNA-1 (miR-1) mediates the induction of pathologic cardiac hypertrophy. Dysfunction of the gap junction protein connexin 43 (Cx43), an established miR-1 target, during cardiac hypertrophy leads to ventricular tachyarrhythmias (VT). However, it is still unknown whether miR-1 and Cx43 are interconnected in the pro-arrhythmic context of hypertrophy. Thus, in this study we investigated whether a reduction in the extent of cardiac hypertrophy could limit the pathological electrical remodeling of Cx43 and the onset of VT by modulating miR-1 levels. Wistar male rats underwent mechanical constriction of the ascending aorta to induce pathologic left ventricular hypertrophy (LVH) and afterwards were randomly assigned to receive 10mg/kg valsartan, VAL (LVH+VAL) delivered in the drinking water or placebo (LVH) for 12 weeks. Sham surgery was performed for control groups. Programmed ventricular stimulation reproducibly induced VT in LVH compared to LVH+VAL group. When compared to sham controls, rats from LVH group showed a significant decrease of miR-1 and an increase of Cx43 expression and its ERK1/2-dependent phosphorylation, which displaces Cx43 from the gap junction. Interestingly, VAL administration to rats with aortic banding significantly reduced cardiac hypertrophy and prevented miR-1 down-regulation and Cx43 up-regulation and phosphorylation. Gain- and loss-of-function experiments in neonatal cardiomyocytes (NCMs) *in vitro* confirmed that Cx43 is a direct target of miR-1. Accordingly, *in vitro* angiotensin II stimulation reduced miR-1 levels and increased Cx43 expression and phosphorylation compared to un-stimulated NCMs. Finally, *in vivo* miR-1 cardiac overexpression by an adenoviral vector intra-myocardial injection reduced Cx43 expression and phosphorylation in mice with isoproterenol-induced LVH. In conclusion, miR-1 regulates Cx43 expression and activity in hypertrophic cardiomyocytes *in vitro* and *in vivo*. Treatment of pressure overload-induced myocyte hypertrophy reduces the risk of life-threatening VT by normalizing miR-1 expression levels with the consequent stabilization of Cx43 expression and activity within the gap junction.

## Introduction

Although cardiac hypertrophy is a compensatory response to overload conditions such as hypertension or valvular heart diseases [1], it is considered an independent cardiovascular risk factor [1,2]. Ventricular tachyarrhythmias (VT), including ventricular fibrillation, are major determinants of sudden death in patients with cardiac hypertrophy [3]. These arrhythmias, in fact, are triggered by the presence of an arrhythmogenic substrate that is typical of hearts with pathological hypertrophy [4]. Several electrophysiological abnormalities have been ascribed to cardiac hypertrophy, including increased duration of action potential, reduced resting membrane potential and delayed conduction velocity (due to interstitial fibrosis). The latter results in depolarization and recovery of heterogeneous areas with increased refractory period [5]. Signaling pathways that promote deterioration of cardiac electrophysiological performance are currently an area of intense research, since their inhibition through fine tuning of the action potential can be considered a new therapeutic approach in arrhythmias [6].

The renin-angiotensin system is up-regulated in response to the stress conditions imposed on the heart [7]. The effects of angiotensin II (AngII) are mainly due to its interaction with the AngII type 1 receptor (AT1R), which belongs to a superfamily of receptors with seven transmembrane domain coupled to G-proteins and whose direct inhibition has been postulated to normalize the electrophysiological properties of cardiomyocytes, probably through the stabilization of their electrical plasmatic membrane. Accordingly, it has been recently shown that AT1R activation could exert arrhythmogenic effect through c-Src tyrosine kinase-mediated connexin 43 (Cx43) displacement from the gap junctions [8]. Cx43 is the highest expressed protein in the myocardium devoted to regulate intercellular connections in mammalian hearts. In normally functioning cardiac ventricles Cx43 is localized at the intercalated disks, where it supports longitudinal and transverse spread of the action potential [9]. The contractile dysfunction and arrhythmias that occur during hypertrophy depend, in addition to a metabolic imbalance, mainly on excitability and electrical coupling. Phosphorylation and dephosphorylation of Cx43 mediated by different protein kinases, affect the processes of trafficking, assembly, degradation, expression, and distribution hence altering the number of functional channels, the so-called "open probability", and conductance properties [10]. Nevertheless, it is not completely understood how Cx43 dysregulation increases arrhythmic risk.

Recent works from our and other laboratories have characterized the role of several microRNAs (miRs) in cardiovascular biology and diseases [11–13]. MiRs are a class of small non-coding RNA molecules, about 22 nucleotides long, which can base-pair sequences partially complementary to the 3'-UTR located in regions of mRNA targets, arresting the process of translation [14]. Selected miRs’ dysregulation alters the cellular responses of cardiomyocytes and non-cardiomyocytes to specific signaling upon the pathological hemodynamic overload, leading to cardiac hypertrophy and heart failure [12]. Furthermore, miRs play an essential role in controlling cardiac excitability since, by acting at post-transcriptional level, such molecules regulate the expression of gap-junctions and ion channels [15,16]. Among the highly expressed miRs in the myocardium, miR-1 has an essential regulatory role in the development of cardiac hypertrophy and it controls cardiac electrophysiology for its ability to modulate the expression levels of molecular targets that adjust the electrical coupling of the cardiac fiber cells [17]. In particular, Cx43 is a direct target of miR-1 gene-silencing activity [34,35]. However, it is still unknown whether miR-1 and Cx43 are interconnected in the pro-arrhythmic context of left ventricular hypertrophy (LVH) and whether miR-1 expression and activity can be regulated by an anti-hypertrophic treatment, such as AT1R antagonization.

Therefore, the purpose of this study was to examine: (i) whether miR-1 and Cx43 dysfunctions underlie the onset of VT associated to cardiac hypertrophy in a rat model of pressure overload; (ii) whether miR-1 directly modulates Cx43 expression and activity in hypertrophic myocytes *in vitro* and *in vivo*; (iii) to assess whether the treatment of pathologic LVH by AT1R blockade could normalize miR-1 levels, limit the adverse electrical remodeling of Cx43 and reduce the induction of life-threatening VT.

## Materials and Methods

### In vivo experimental protocols

Male Wistar rats (90-120g, 7 weeks of age, N=22) were used for an aortic banding protocol conformed to Directive 2010/63/EU of the European Parliament and approved by the Italian Ministry of Health and by Institutional Animal Care and Use Committee of Magna Graecia University. Briefly, rats were anesthetized as previously reported [18], and after confirmation of pain reflex absence, were intubated and ventilated by dedicated apparatus while placing a tantalum clip on the ascending aorta through a left lateral mini-thoracotomy; in further twenty rats a *sham* surgery was performed. After allowing recovery, animals were randomized to receive either placebo (LVH, n=12; SHAM, n=10) or valsartan at the dosage of 10 mg/kg/day added to the drinking water (LVH+VAL, n=10; SHAM+VAL, n=10) for 12 weeks.

In an additional set of experiments to assess the direct role of miR-1 on Cx43 expression and activity, myocyte hypertrophy was induced in 8-12 weeks old C57BL/6 mice by Isoproterenol daily injection (Iso, 50mg/kg body weight i.p.) for 14 days [19]. Briefly, in a group of mice (n=7) an Adenoviral vector (10^11^ pfu/mL) carrying a miR-1 construct under the ubiquitous CMV promoter (Ad-miR-1) [11] was intra-myocardially released by 5 direct epicardial injections of 3µL each in the anterior LV and apical region, followed by delivering of 30µL of adenoviral construct dissolved in 30% pluronic F127 gel (Sigma), in order to cover the entire LV wall. An Adeno-Empty vector was equally injected in additional mice (n=7). Levels of miR-1 are significantly up-regulated in cardiac muscle already at 48-72 hours (*data not shown*). Thus, 72 hours later, 8 adenovirus-transfected mice were administered Iso as above described (n=4, Ad-Empty+Iso and n=4, Ad-miR-1+Iso) while 6 mice were administered only saline solution (n=3 Ad-Empty+Saline and n=3 Ad-miR-1+Saline). Mice were accordingly sacrificed 14 days later [19].

At the end of the study protocol, each rat or mouse was sacrificed by lethal anaesthesia and morphometric parameters were analyzed. Subsequently, LV was longitudinally cut in two halves and one of the two was flash-frozen in liquid nitrogen and stored at -80°C for molecular biology studies while the other was fixed in 10% buffered formalin for immunohistochemistry studies (see below).

### Echocardiographic evaluation

Echocardiographic measurements were obtained in basal conditions and after 12 weeks from banding. Briefly, under light anesthesia, examinations were performed with a 7.5MHz phased-array transducer (VIVID E, GE Healthcare, Fairfield, CT, USA), as previously described [20]. Interventricular septum and posterior wall thicknesses (IVSWth and PWth, respectively), LV end-systolic (LVESD) and end-diastolic (LVEDD) diameters were measured with standard technique in parasternal long and short axis. Finally, the fractional shortening (FS) was calculated by two blinded observers.

### Hemodynamic studies

A 2F conductance catheter (Millar Instruments Inc, Houston, TX, USA) was inserted retrogradely through the right common carotid artery into the LV 12 weeks after aortic banding. Systolic blood pressure (SBP) and diastolic BP (DBP) were recorded at level of the aortic arch, then catheter was advanced through the clip for trans-stenotic supravalvular gradient assessment (in banded rats), and finally heart rate (HR), LV end diastolic pressure (LVEDP), LV maximal systolic pressure (LVmax) and the maximal and minimal first derivative of the LV pressure over time [LV(dP/d*t*_max_) and LV(dP/d*t*_min_), respectively], were assessed at intracavitary level [21]. Data were acquired on digital media at a sampling of 1,000Hz (PowerLab System, ADInstruments Inc, Colorado Springs, CO, USA) and all the parameters were analyzed offline [22] by a dedicated software (LabChart, ADInstruments Inc, Colorado Springs, CO, USA).

### Electrophysiological study

Electrophysiological evaluation was performed at the end of the study after hemodynamic assessments in rats of each group. A stable 3-lead electrocardiographic digital tracing was obtained by placing the leads in standard position not interfering with other equipments [23] and a dedicated bipolar catheter was advanced anterogradely into the right ventricle through the external jugular vein. A standard intracavitary stimulation protocol of twenty impulses at a cycle length of 100ms (S_1_), followed by 3 extrastimuli (S_2_, S_3_, and S_4_) at shorter coupling intervals was delivered at 2V output energy and 0.5ms pulse width by using an external apparatus (MEDICO, Italy). Ventricular capture was assessed online while the induced ventricular arrhythmias were offline analyzed using LabChart7Pro software (ADInstruments Inc, Colorado Springs, CO, USA).

### Recording of intracavitary electrograms

Electrograms were recorded on a multichannel computerized data acquisition system at a sampling rate of 1,000Hz. At this sampling rate, temporal resolution was sufficient for measuring rapid electrical deflections over short distances. The ventricular effective refractory period (VERP), deﬁned as the longest coupling interval of the premature stimulus that failed to activate the entire heart, was determined at the time of electrophysiological studies. Signals were band-pass filtered (low cutoff, 0.16Hz; high cutoff, 1kHz) and digitized with 16bit resolution and a sampling frequency of 1kHz. The selected gain for balancing input noise of the system was 4µV (peak-peak). Every twentieth stimulus was followed by 1 premature stimulus. Starting at basal cycle length of 150ms, the coupling interval of the premature stimulus was reduced in steps of 5ms until VERP [24]. Monophasic action potential duration at 90% repolarization (MAPD90) was evaluated at endocardial level. Signals were amplified, band-pass filtered, and digitized by a dual bioamplifier (ADInstruments) before extraction and analysis. Recordings were accepted if they had a stable baseline, a rapid upstroke phase with consistent amplitude, a smooth repolarization phase, and a stable duration. MAPD90 was defined as the monophasic action potential interval from the onset of zero-phase depolarization to the 90% repolarization level. Basically, right ventricles were stimulated as above indicated for VERP and activation was assessed as previously described [24].

### Tissue Harvesting, Histology, Immunohistochemistry and Confocal Microscopy

After completion of the cardiac function measurements, the isolated hearts were cut into right and left ventricles, and right and left atria. After being weighed, the LV was sectioned into 2 parts, and one of the two was fixed in 10% formalin and paraffin embedded, and 5µm cross sections were prepared on a microtome (Leica). Mouse cardiomyocyte cross-sectional area was obtained as previously described [19]. Immunohistochemistry and confocal microscopy were performed as previously described [25,26]. Briefly, myocyte cytoplasm was detected using an antibody against α-sarcomeric actin (1:50 dilution; clone 5C5, Sigma), for 2hrs at 37°C and this was detected with anti-mouse IgM Texas Red (1:100 dilution; Jackson Immunoresearch). Cx43 was detected with a rabbit polyclonal antibody against Cx43 (1:50 dilution; Abcam) overnight at 4°C. This antibody was detected with an anti-rabbit IgG FITC (1:100 dilution; Jackson Immunoresearch). Cx43 phosphorylated on Ser 279/282 was detected with a goat polyclonal (1:50 dilution; Santa Cruz) antibody for 2hrs at 37°C which was ligated by an anti-goat IgG FITC (1:100 dilution; Jackson Immunoresearch). Nuclei were counterstained with DAPI. Sections were mounted in Vectashield, analyzed and scanned using confocal microscopy (Zeiss LSM 710).

### Isolation and culture of neonatal rat ventricular cardiomyocytes

Primary cultures of cardiomyocytes were obtained from both ventricles of rats at 1-2 days of age. Isolated ventricles were plated in D–PBS with heparin, minced and enzymatically digested. Fragments were placed in a solution combining trypsin (Gibco-Life Technologies Italia, MB, Italy) and DNAse I (Sigma Aldrich, St. Louis, MO, USA) in MEM base and subjected to several cycles of digestion at 37°C, then placed in flasks pre-treated with laminin and resuspended in D-MEM with 10% FBS. After 48hrs the culture medium was replaced with medium containing supplements and 3% FBS [27]. On the third day cardiomyocytes were stimulated with 5µmol/L AngII or AngII plus 10µmol/L VAL for 3hrs. Cells were then collected and evaluated for immunoblotting analysis and Real Time RT-PCR quantification.

### Ca^2+^ transients

The intracellular Ca^2+^ transients were measured in basal conditions and after AngII treatment alone or in combination with VAL, as previously described [28]. Briefly, fluo-3 dye was added to cultured cells after respective treatments directly into DMEM (10µM for 15min), then fluorescence was assessed at confocal laser microscopy imaging. Acquisitions were processed to obtain a peak value of F/F_0_, whereas F is the peak of fluorescent level and F_0_ is the resting fluorescent level.

### Determination of RNA levels by RT-PCR

Extracts of total RNA were obtained from both LV and cultured cardiomyocytes of each group using mirVana miRNA Isolation Kit (Ambion-Life Technologies Italia, MB, Italy) according to the manufacturer’s protocol. Expression levels of mature miRNAs [29] were analyzed by real-time RT-PCR using TaqMan microRNA assays (Applied Biosystems, Foster City, CA). In brief, 10ng of total RNA were reverse-transcribed with specific stem-loop RT primers using TaqMan microRNA reverse transcription kit, according to the manufacturer’s instructions. Real-time RT-PCR was performed on cDNA using specific primers designed on rat miR-1 sequence 5'-UGG AAU GUA AAG AAG UGU GUA U-3'. The reactions were incubated in a 96-well plate at 95°C for 10min, followed by 40 cycles at 95°C for 15s followed by 60°C for 60s. The U6 expression of the housekeeping gene was used as endogenous control for data normalization. Cx43 mRNA levels were assessed by Taqman-gene expression assay [13].

### Preparation of protein extracts and membrane fractionation

Rat hearts were homogenized in a buffer consisting of Tris/EDTA (Tris/HCl 50mM, EDTA 4mM, pH 7.4), Triton X-100 and protease and phosphatase inhibitors. After homogenization, lysates were kept on ice for 20min and then centrifuged at 2000 rpm for 5min at 4^°^C. Supernatant was then transferred to high speed tubes and centrifuged again at 17.500 rpm for 25min at 4^°^C; finally, supernatant (cytosolic fraction) was isolated and pellet (membrane fraction) was resuspended in 200µL buffer [22]. Protein concentrations were determined by standard technique. After SDS-PAGE separation, proteins were transferred to nitrocellulose membranes and incubated with specific antibodies for Akt, ERK1/2, Cx43. Specific HRP-conjugated secondary antibodies were used according to the manufacturer’s recommendations (Santa Cruz Biotechnology-DBA Italia, MI, Italy). Specific protein bands were detected by chemiluminescence using the Chemidoc XRS system (Bio-Rad Laboratories, MI, Italy).

### Statistical analysis

Data analysis was performed using analysis of variance (ANOVA) with SPSS10.0 software (SAS Institute Inc., Cary, NC, USA). When a significant overall effect was found, Bonferroni test was used to compare mean values. Values of p<0.05 were considered statistically significant.

## Results

### AT1R Blockade Improves LV performance and Inhibits Detrimental Pathologic Molecular Adaptations by Reducing LVH

**Figure 1:**
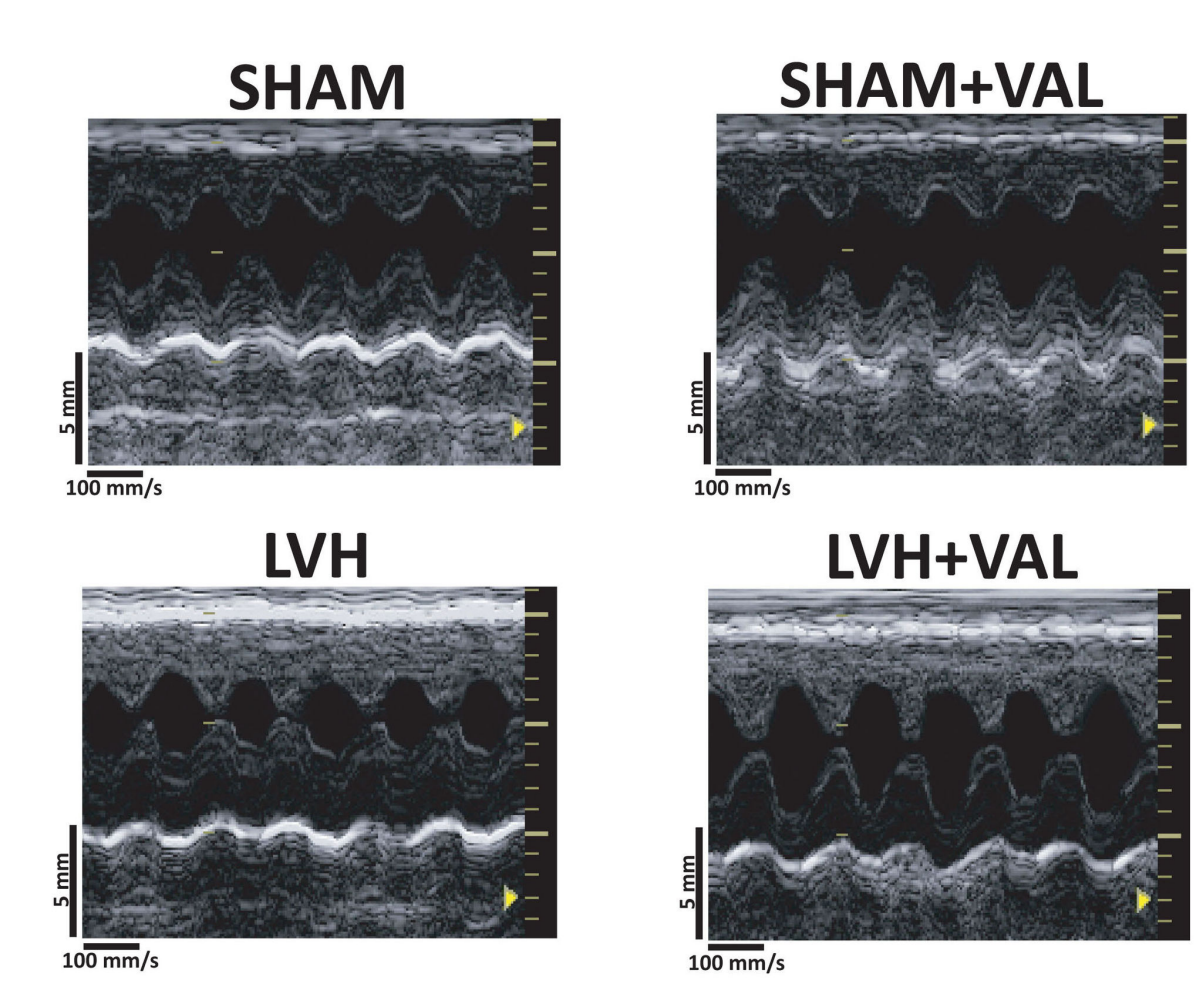
Representative echocardiographic acquisitions from each group of rats. Two-dimensional parasternal long axis images demonstrating increase in wall thickness after ascending aorta banding (LVH) compared to control groups (SHAM, top left panel; SHAM+VAL, top right panel) and reduction in the extent of hypertrophy after chronic valsartan administration (LVH+VAL). Depth: 5mm, digital acquisition speed: 100mm/s.

Ascending aorta banding determined LVH after 12 weeks, as indicated by morphometric measurements (28.6% increase in left ventricular weight/body weight, LVW/BW; 32.7% increase in heart weight/body weight, HW/BW, p<0.05 vs. all) and echocardiographic parameters (26.1% increase in IVSWth; 25.9% increase in PWth, p<0.05 vs. all) when compared to sham-operated animals. Chronic treatment with VAL significantly reduced LVH (LVW/BW=1.92±0.12; HW/BW=2.53±0.13; IVSWth=1.47±0.08; PWth=1.35±0.06, Table 1, and Figure 1).

**Table 1:** Morphometric and echocardiographic parameters from *sham-operated* and *pressure-overloaded* rats treated with Valsartan or placebo for 12 weeks after surgery.

	SHAM (N=10)	SHAM+VAL (N=10)	LVH (N=12)	LVH+VAL (N=10)
BW, g	352.2 ± 8.7	350.3 ± 14.2	345.2 ± 18.2	351.6 ± 17.5
LVW, mg	639.4 ± 7.8	638.3 ± 12.8	817.5 ± 10.9*	667.4 ± 8.9
RVW, mg	138.6 ± 8.4	137.3 ± 8.2	152.8 ± 7.7*	130.6 ± 8.7
AW, mg	49.6 ± 5.3	53.0 ± 6.1	86.0 ± 9.3*	82.3 ± 8.9
HW, mg	827.6 ± 0.5	828.6 ± 0.8	1056.3 ± 0.12*	880.3 ± 0.13
LVW/BW, mg/g	1.84 ± 0.02	1.85 ± 0.01	2.38 ± 0.09*	1.92 ± 0.12
HW/BW, mg/g	2.32 ± 0.05	2.31 ± 0.08	3.08 ± 0.11*	2.53 ± 0.13
LW, mg	1243.2 ± 59.7	1236.8 ± 64.0	1308.5 ± 55.0	1305.4 ± 49.6
LVEDD, mm	4.90 ± 0.28	4.87 ± 0.27	4.79 ± 0.37	4.94 ± 0.35
LVESD, mm	2.18 ± 0.10	2.22 ± 0.26	2.72 ± 0.26	2.50 ± 0.24
IVSWth, mm	1.40 ± 0.07	1.38 ± 0.06	1.74 ± 0.20*	1.47 ± 0.08
PWth, mm	1.39 ± 0.07	1.38 ± 0.13	1.75 ± 0.12*	1.35 ± 0.06
FS, %	55.5 ± 1.9	54.3 ± 4.3	43.2 ± 2.4*	49.5 ± 3.1

Values are expressed as mean ± SEM. SHAM, rats undergone left thoracotomy; SHAM+VAL, rats undergone left thoracotomy followed by administration of 10mg/kg/die Valsartan; LVH, rats undergone left thoracotomy and aortic banding with tantalum clip; LVH+VAL, rats undergone left thoracotomy and aortic banding with tantalum clip followed by administration of 10mg/kg/die Valsartan; BW, body weight; LVW, left ventricular weight; RVW, right ventricular weight; AW, left plus right atria weight; HW, whole heart weight; LW, lungs weight; LVEDD, left ventricular end-diastolic diameter; LVESD, left ventricular end-systolic diameter; IVSWth, interventricular septum wall thickness; PWth, posterior wall thickness; FS, fractional shortening. Analysis of variance for unpaired data was used to analyze results. *p<0,05 vs. all.

**Figure 2:**
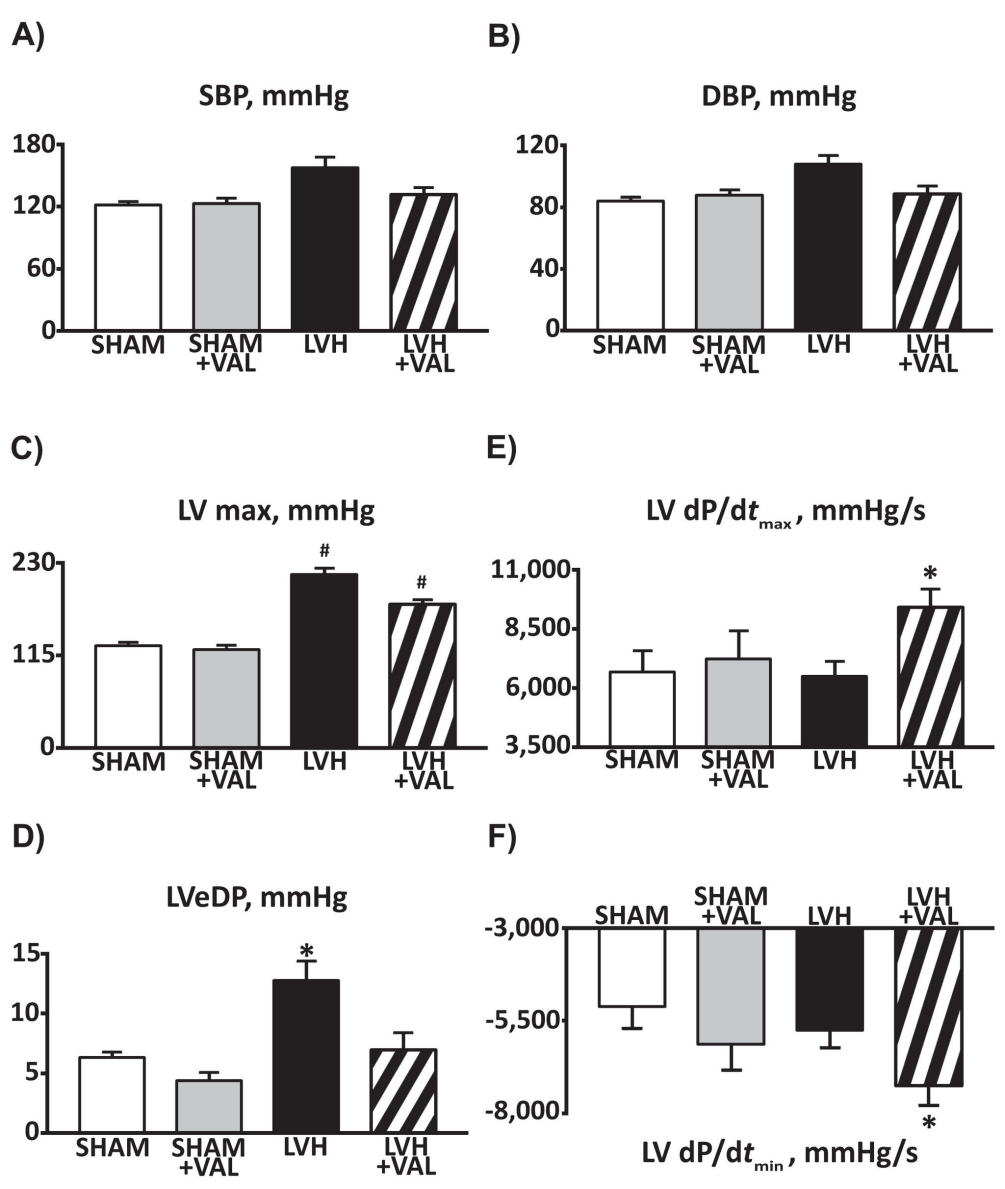
Cumulative data on hemodynamic findings from sham-operated and pressure-overloaded rats. **A**: Valsartan administration improved systolic and diastolic (**B**) profiles (systolic blood pressure and diastolic blood pressure, SBP and DBP, respectively) in hypertrophied rats. **C**: Banding of the ascending aorta determined a significant increase in left ventricular maximal pressure (LV max), whereas LV end-diastolic pressure (LVeDP, panel **D**) was significantly higher in hypertrophic hearts compared to both sham groups and Valsartan-treated hypertrophic hearts. **E**, **F**: The maximal and minimal first derivative of the LV pressure over time [LV(dP/d*t*_max_) and LV(dP/d*t*_min_), respectively] demonstrated improved cardiac function in rats treated with Valsartan for twelve weeks after aortic banding (*p<0.03 vs. all; ^#^ p<0.05 vs. SHAM and SHAM+VAL).

Hemodynamic studies, performed 12 weeks after banding, demonstrated normal systemic pressure values (both systolic and diastolic, Figure 2A and 2B) among the four groups; as expected, aortic banding was associated to an increased LV pressure (216.0±22.3 mmHg for LVH group; 178.8±13.2mmHg for LVH+VAL group, compared to sham-operated animals (p<0.01 vs. SHAM and SHAM+VAL, Figure 2C), whereas LVEDP was found elevated only in the LVH group (12.8±5.6mmHg, p<0.01 vs. all, Figure 2D). Furthermore, contractile function was assessed by measuring the maximal and minimal first derivative of the LV pressure over time [LV(dP/d*t*_max_) and LV(dP/d*t*_min_), respectively, Figure 2E and 2F] demonstrating increase in both systolic (dP/d*t*_max_, 9432.9±2259.7 mmHg/s) and diastolic (dP/d*t*_min_, -7240.1±1591.3 mmHg/s) functions in LVH+VAL compared to LVH and to sham-operated controls. Finally, trans-stenotic systolic pressure gradient, calculated as difference between supravalvular systolic pressure and systolic blood pressure, was confirmed in rats subjected to ascending aorta banding (LVH= 60.1±22.1mmHg; LVH+VAL= 53.4±24.6mmHg, p=NS), indicating a superimposable stress to hearts of both banded groups along the entire duration of the study.

**Figure 3:**
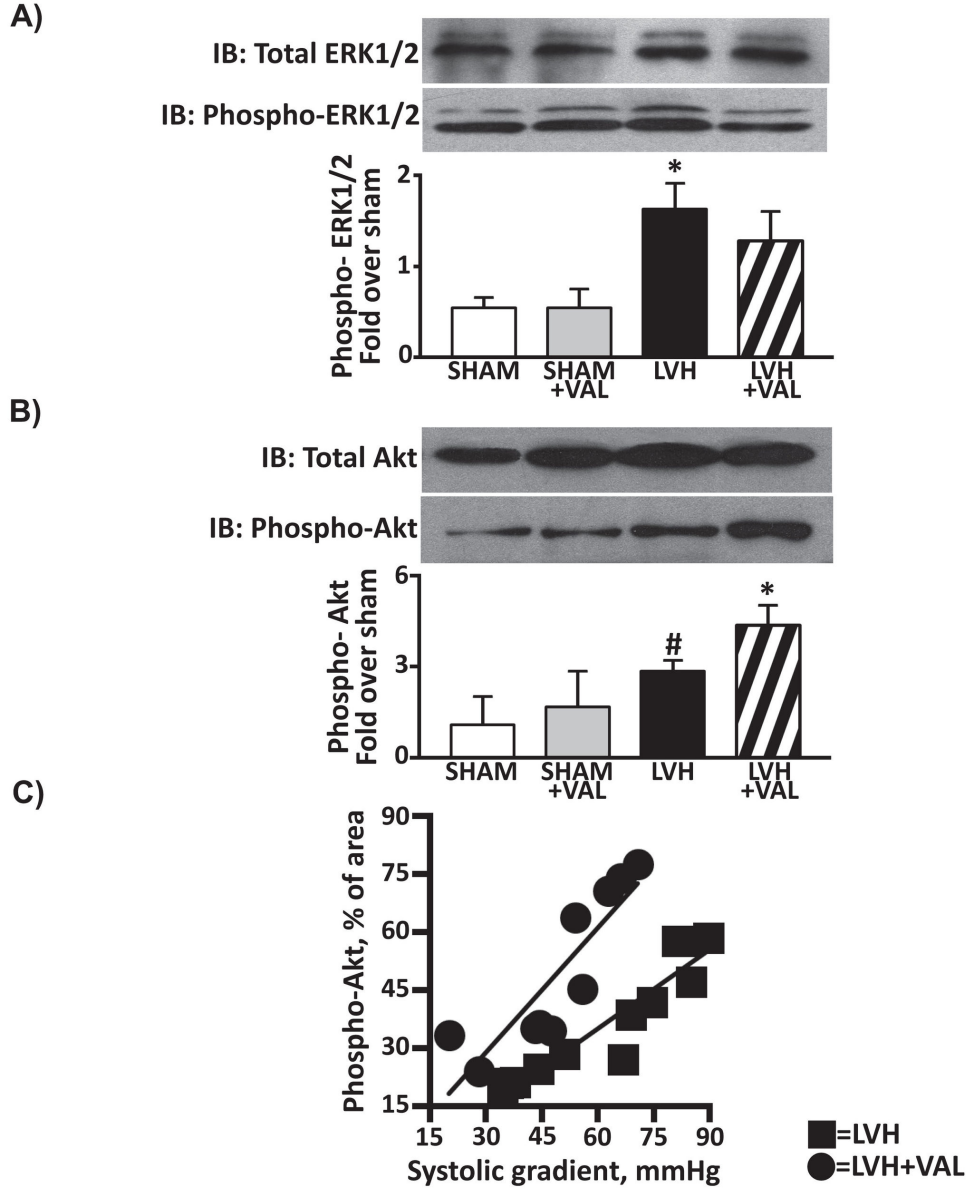
The maladaptive and detrimental molecular activation in hypertrophic hearts is attenuated by AT1R blockade. **A**: Increased phosphorylation of MAPK-ERK1/2 was observed in LVH group (1.6 fold over sham) compared to sham groups and to LVH+VAL group (1.3 fold over sham, *p<0.05 vs. all). **B**: Cardiac levels of activated Akt were significantly higher twelve weeks after aortic banding compared to sham-operated rats (2.8 fold over sham, ^#^ p<0.05 vs. SHAM and SHAM+VAL), and were even more increased in hypertrophic group treated with valsartan (4.4 fold over sham, *p<0.05 vs. all). **C**: Correlation between trans-stenotic systolic supravalvular gradient and activation of Akt in hypertrophied hearts; untreated rats with aortic banding (squares, LVH, N=12) show reduced levels of p-Akt over the entire spectrum of systolic gradients (20.1÷89.6mmHg) compared to hypertrophied rats treated with valsartan (circles, LVH+VAL, N=10).

Accumulating evidences strongly implicate AngII intracellular signaling in mediating pathologic cardiac remodeling and failure [6,30]. AngII activates various intracellular protein kinases, and among them the serine/threonine kinases of the mitogen-activated protein kinase (MAPK) family, and the Akt/protein kinase B play a key role in mediating AngII/AT1R effects. Thus, total and phosphorylated (activated) levels of ERK1/2 and Akt were investigated in order to assess the signaling pathways downstream AT1R. A three-fold increase in p-ERK1/2 was observed in LVH group compared to SHAM (1.6±0.3 vs. 0.5±0.7, p<0.05), and a similar increase was detected in LVH+VAL vs. SHAM+VAL (2.4 fold increase,1.3±0.3 vs. 0.5±0.2, p<0.05), with ~30% difference between LVH and LVH+VAL (Figure 3A). On the other hand, aortic banding determined a significant increase in phospho-Akt levels twelve weeks after development of hypertrophy compared to controls both in placebo groups (1.1±0.9 vs. 2.8±0.3, SHAM vs. LVH, p<0.01) and in valsartan groups (1.7±1.1 vs. 4.4±0.6, SHAM+VAL vs. LVH+VAL, p<0.01). Interestingly, valsartan treatment in aortic banded rats produced a 54% increase in p-Akt when compared to the untreated aortic banded hypertrophic group (p<0.05, Figure 3B). Therefore, a differential activation of ERK1/2 and Akt was observed upon banding in the LVH group compared to the LVH+VAL group.

A sustained benefit from reducing the extent of hypertrophy was observed over the entire range of chronic pressure overload (20.1÷89.6mmHg, trans-stenotic systolic pressure gradient) as it was plotted against the entire range of activated Akt (14.4÷77.6 arbitrary unit, p-Akt area, Figure 3C) indicating that the administration of the AT1R inhibitor in animals with cardiac pressure overload induced a significant increase in cardiac protective pro-survival signaling.

### Left Ventricular Hypertrophy is associated with increased susceptibility to ventricular tachyarrhythmias

**Figure 4:**
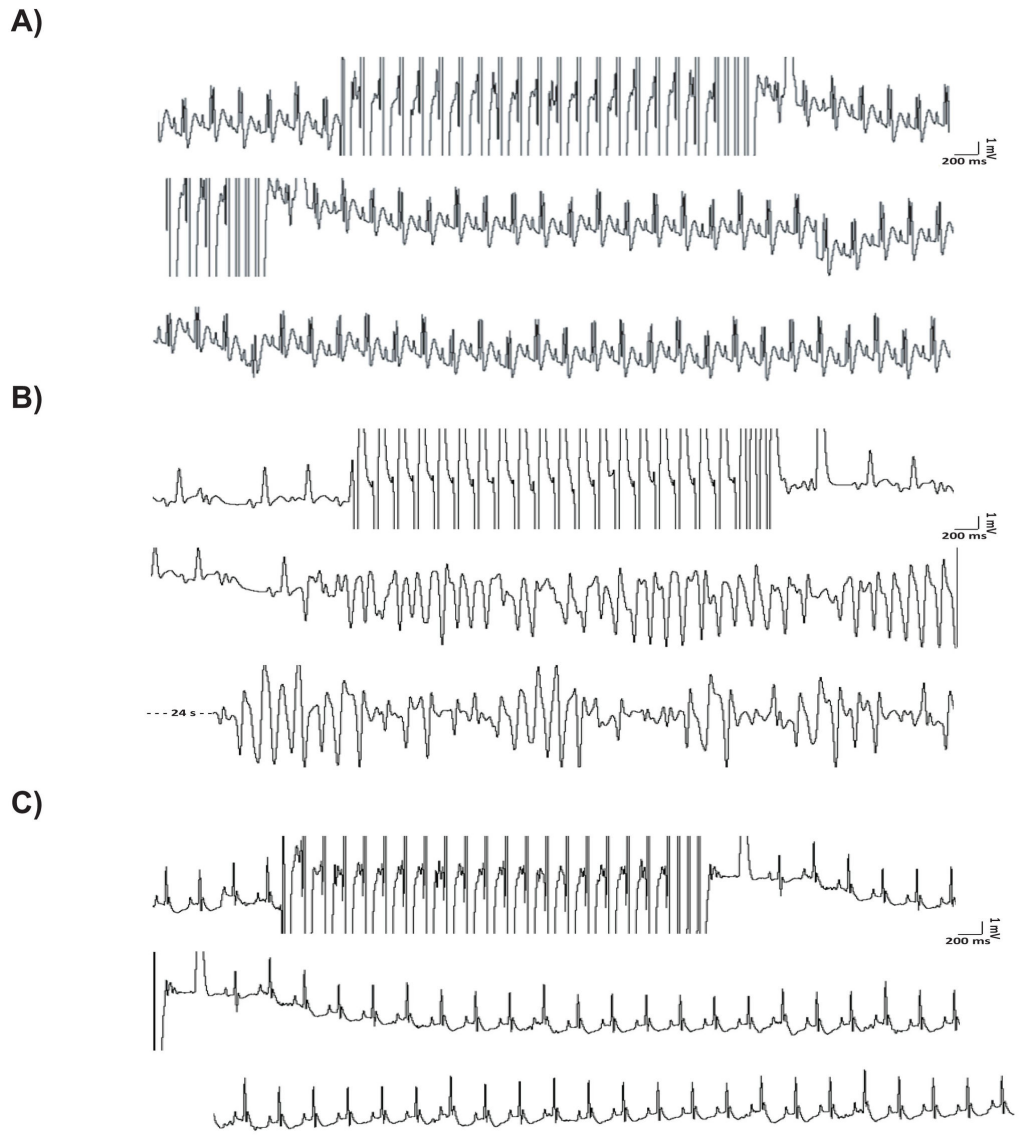
Susceptibility to the onset of ventricular tachyarrhythmias assessed during programmed electrical stimulation. **A**: Programmed electrical stimulation (PES) was performed with a dedicated bipolar catheter, which was advanced anterogradely into the right ventricle. A train of N=20 impulses at 100ms of basal cycle length followed by three extrastimuli at 50ms was used in each case; no arrhythmias were inducible in rats from neither SHAM nor SHAM+ VAL groups. **B**: R-on-T phenomena before and after stimulation in hypertrophied rat hearts with the induction of a sustained ventricular tachycardia at the end of stimulation. **C**: In contrast, after valsartan administration only ventricular extra beats were inducible in hypertrophied rats in which stable sinus rhythm was restored at the end of stimulation. Central strips from each panel start from previously recorded 1000ms of respective upper strips, as for bottom strips from panels A and C, whereas bottom strip from panel B is postponed of 24 seconds due to induction of sustained ventricular tachycardia.

**Figure 5:**
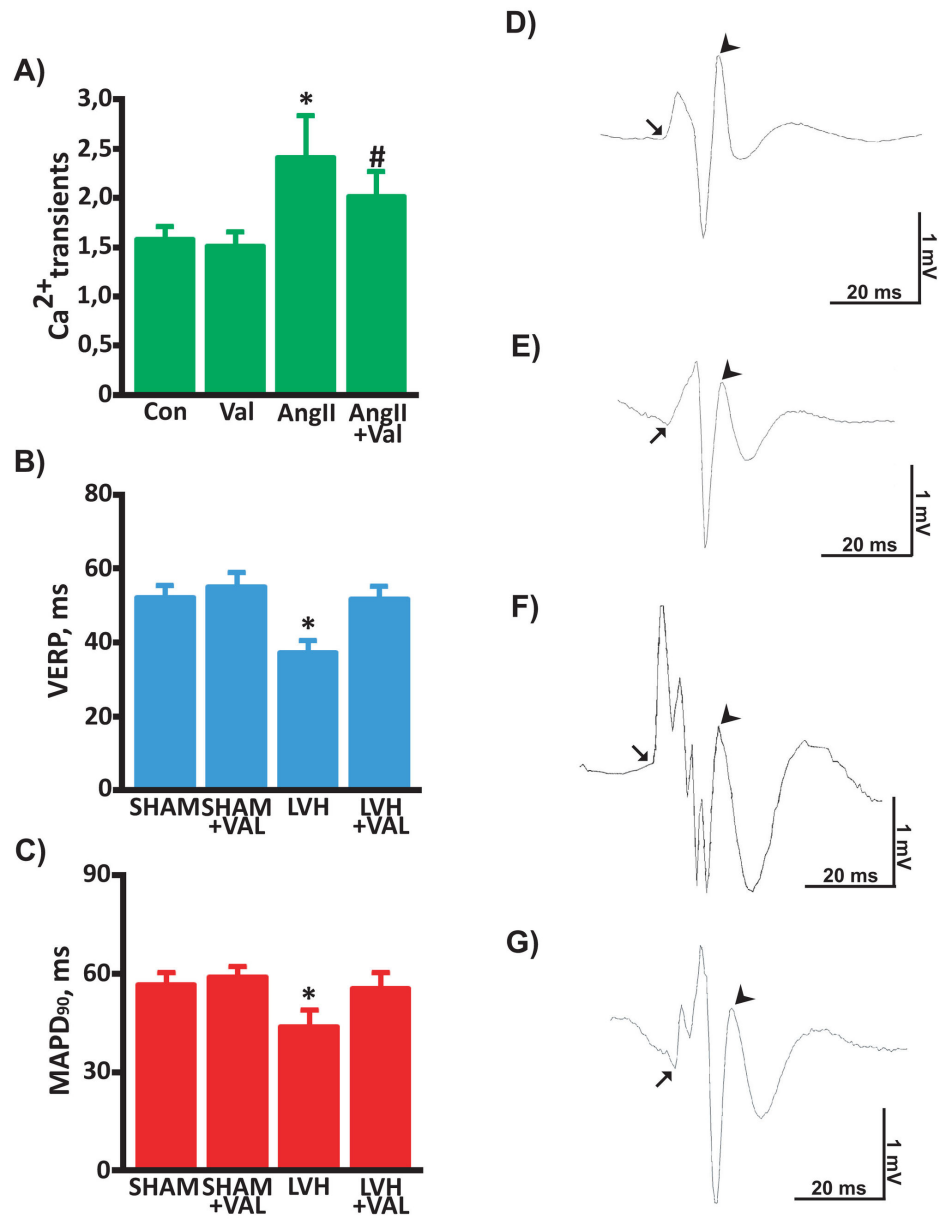
Valsartan modulation of electrophysiological properties *in* *vitro* and *in* *vivo*. **A**: Bar graph demonstrating average peak systolic amplitude of the calcium transients, expressed as absolute numbers from ratio between the peak of fluorescence intensity (*F*) and the intensity at rest (F_*0*_): AngII stimulation (10^-6^mol/L; 24 hours) augmented transients in cultured myocytes, which were attenuated by the AngII type 1 receptor blocker valsartan. Valsartan alone did not change the amplitude of calcium transient. The mean peak F/F_0_ was significantly higher in AngII–treated cells than control cells (*p<0.05 vs. all, ^#^ p<0.003 vs. Con and AngII). **B**: Ventricular effective refractory period (VERP) was dramatically reduced in LVH (37.3±3.4ms) compared to SHAM (52.3±2.6ms), SHAM+VAL (55.1±3.3ms), and LVH+VAL (51.9±2.9ms, *p<0.001 vs. all). **C**: Monophasic action potential duration at 90% repolarization (MAPD_90_) was significantly shortened in hypertrophic hearts (LVH= 44.0±4.3ms) as opposed to control groups (SHAM= 56.7±2.9ms; SHAM+VAL= 59.1±2.5ms), whereas valsartan significantly prolonged it (LVH+VAL= 55.6±4.3ms *p<0.001 vs. all). **D** through **G**: Representative endocardial ventriculograms recorded from the right ventricles indicating normal myocardial conduction in SHAM (panel D) and in SHAM+VAL (panel E), whereas conduction dispersion was observed in LVH (panel F) and LVH-VAL (panel G), the latter showing same duration in ventricular activation compared to SHAM and SHAM+VAL. Arrows indicate onset of ventricular depolarization, arrow-heads indicate end of ventricular depolarization, followed by abnormal repolarization in hypertrophic hearts, which was more pronounced in LVH (panel F, see Table 2 for data).

Basal electrocardiographic acquisitions in anesthetized rats did not display any difference in HR among the groups (Table 2). Of note, an increased number of premature ventricular contractions (PVC) was observed in LVH vs. SHAM group (1200±180counts/hr vs. 255±165counts/hr, p<0.03 vs. all). These PVCs were mostly characterized by R-on-T phenomena and were detected before starting the electrophysiologic study. Interestingly, the reduction in the extent of cardiac hypertrophy by Valsartan was associated with a decrease in PVC counts (LVH+VAL= 650±200counts/hr, p<0.05 vs. SHAM and SHAM+VAL). The intracavitary stimulation performed after 12 weeks did not trigger VT in sham-operated control rats (Figure 4A); on the contrary, provocative invasive study displayed an increased incidence of VT in hypertrophied rats compared to sham rats (8/12, 66.7%, Figure 4B). The arrhythmic vulnerability, represented by susceptibility to ventricular fibrillations and/or sustained ventricular tachycardias at the end of electrophysiologic study, was dramatically reduced in hypertrophic rats treated with VAL (1/10, 10%, p<0.006, Figure 4C). In order to provide overall mechanistic effect of arrhythmogenesis, spontaneous calcium transients were assessed in cultured cardiomyocytes. In fact, it has been recently reported that AngII enhances L-type calcium channel current in cardiomyocytes during depolarization [31] and thereby increases intracellular calcium loading, which may result in triggered activity and arrhythmia. To this regard, calcium transient monitoring demonstrated increased levels upon AngII stimulation (2.4±0.4) compared to control cultured cardiomyocytes (1.6±0.1, Figure 5A) which were reduced by valsartan administration (Val= 1.5±0.1; AngII+Val= 2.0±0.2, *p<0.05 vs. all, ^#^ p<0.003 vs. Con and AngII). Moreover, VERP (Figure 5B) and MAPD_90_ (Figure 5C) were significantly reduced in hypertrophic hearts, whereas valsartan treatment was associated to restoration at basal values of both parameters. Finally, the intracavitary electrophysiological study provided additional data on conduction velocity, since QRS complexes were significantly wider (Table 2, and Figure 5, D through G) in LVH compared to LVH+VAL and to sham-operated rats.

**Table 2:** Surface electrocardiographic parameters, intracavitary electrograms recordings, and electrophysiological properties of endocardial cells in *sham-operated* and in *pressure-overloaded* rats treated with Valsartan or placebo for 12 weeks after surgery.

	SHAM (N=10)	SHAM+VAL (N=10)	LVH (N=12)	LVH+VAL (N=10)
HR, bpm	437±38	434±49	399±61	392±49
RR interval, ms	138±11	140±16	155±33	155±19
PVC, counts/hr	255±165	360±162	1200±180*	650±200^#^
QRS duration, ms	11.2±0.8	11.4±1.0	15.1±1.3*	12.0±0.8
QRS amplitude, mV	2.0±0.1	2.0±0.2	3.2±0.3*	2.8±0.2^#^

Values are expressed as average ± SEM. SHAM, rats undergone left lateral thoracotomy only; SHAM+VAL, rats undergone left lateral thoracotomy and treated with valsartan 10 mg/kg/die; LVH, rats undergone aortic banding; LVH+VAL, rats undergone aortic banding and treated with valsartan 10 mg/kg/die; HR, heart rate; bpm, beats per minute; RR interval, length between two consecutive ventricular depolarizations; PVC, premature ventricular contractions; ms, milliseconds; mV, milliVolts. Analysis of variance has been used in data analysis. *p<0,05 vs. all; ^#^p<0,05 vs. SHAM and SHAM+VAL.

### Cx43 expression and phosphorylation is altered upon hypertrophic stress in vitro and in vivo

Cx43 is phosphorylated at its C-terminus residues (S255/S262/S279/S282) by MAPKs altering gap junction formation [32]. This activation leads to a sharp modification of the pore-channel function, resulting in a conformational change that reduces the permeability and conductance of the channel. This phenomenon implies an overall reduction in conduction velocity and greater global dispersion of action potential and refractoriness, maintaining an arrhythmogenic substrate [33].

**Figure 6:**
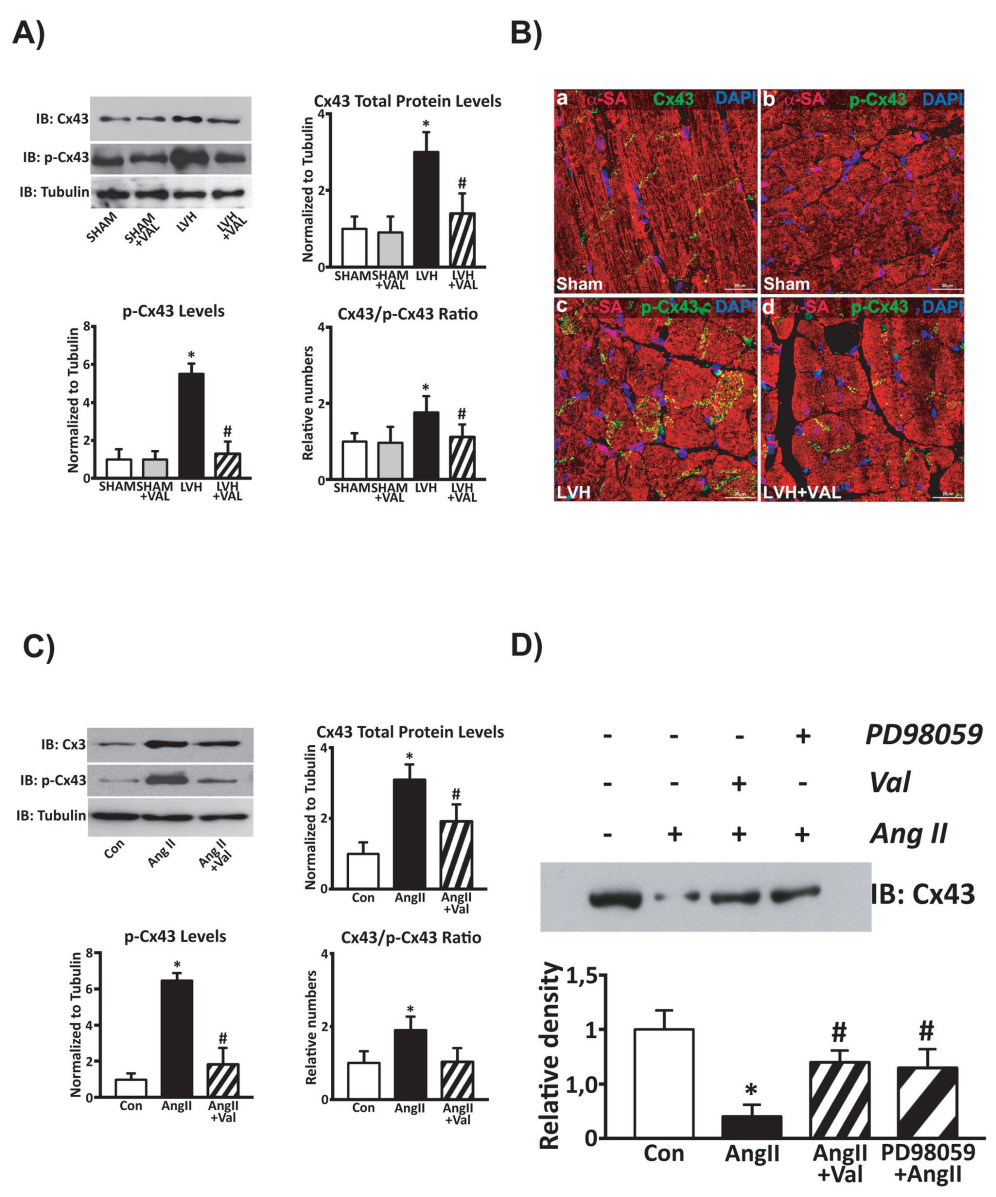
Phosphorylation at residues Ser279/Ser282 is associated with connexin 43 displacement. *In vivo* (A) and *in vitro* (C) phosphorylated levels of Connexin 43. **A**: An increased Cx43 total protein expression along with its hyperphosphorylation at Ser279/Ser282 was observed in cardiac lysates from hypertrophic hearts compared to SHAM and SHAM+VAL, while chronic administration of Valsartan reduced both these two molecular responses; *p<0.05 vs. Sham; #p<0.05 vs. LVH. **B**: high-magnification immunohistochemistry and confocal representative images (*red*: α-sarcomeric actin, α-SA; *green*: Cx43 or p-Cx43; *blue*: DAPI) show normal Cx43 localization within gap-junction (panel *a*) and the very little, if not negligible, levels of Ser279/Ser282-phosphorylated Cx43 (panel *b*) in rat SHAM hearts. LVH by pressure overload is associated with an intense Cx43 phosphorylation at Ser279/282 and its displacement from gap junction (panel *c*). The latter is significantly attenuated by VAL treatment (panel *d*). **C**: Increased protein levels and hyper-phosphorylation of Connexin 43 in cardiomyocytes treated with Angiotensin II (AngII, 5µmol/L) compared to un-stimulated cardiomyocytes, with a significant reduction after concurrent valsartan administration (10µmol/L, *p<0.05 vs. Con; #p<0.05 vs. AngII). **D**: Representative western blot of Cx43 total protein levels in lysates from membrane fractions of cultured cardiomyocytes. After AngII challenge, Cx43 was displaced from the gap junction as demonstrated by its reduced levels in the membrane fractions (lane 2) compared to un-stimulated cells, whereas Valsartan (lane 3) and the MAPK/ERK1/2 kinase inhibitor PD98059 (lane 4) both reduced Cx43 gap junction displacement in AngII-treated myocytes.

*In vivo* Cx43 expression and its Ser 279/282-phosphorylation were significantly increased in LVH group compared to sham-operated rats (p<0.05 vs. all) whereas chronic VAL administration in hypertrophied rats determined a significant decrease of both total and phosphorylated Cx43 in cardiac lysates (Figure 6A). Normalization of Ser 279/282-phosphorylation to total Cx43 protein levels shows that this post-translation modification was modulated by LVH on top of the increased protein expression and thus VAL reduced both Ser 279/282-phosphorylation and total Cx43 protein levels (Figure 6A). Importantly, immunohistochemistry and confocal microscopy revealed that Ser 279/282-phospho-Cx43 was dispersed away from the intercalated disks of cardiomyocytes in the LVH group. Accordingly, valsartan treatment reduced the amount of phospho-Cx43 within cardiomyocyte cytoplasmic compartments (Figure 6B).

Concurrently, immunoblotting analysis on cultured cardiomyocytes revealed a 3-fold increase of Cx43 protein expression associated with a marked Ser 279/282-phosphorylation (>6 fold over control) upon challenge with AngII 5µmol/L stimulation for three hours compared to control cardiomyocytes (Figure 6C). Concomitant *in vitro* stimulation with VAL (10µmol/L) significantly prevented AngII-induced increase in Cx43 protein levels and its hyper-phosphorylation (Figure 6C). AngII-induced Cx43 hyper-phosphorylation determined a displacement of Cx43 from the gap junction as demonstrated by a decreased amount of total Cx43 protein levels in the myocyte membrane fraction when compared to untreated normal myocytes. Intriguingly, Valsartan and the MAPK/ERK1/2 kinase inhibitor (PD98059) both increased Cx43 expression within the myocyte membrane and consequently in the gap junction in AngII-treated myocytes (Figure 6D).

### miR-1 modulates Cx43 expression and phosphorylation in hypertrophic cardiomyocytes

**Figure 7:**
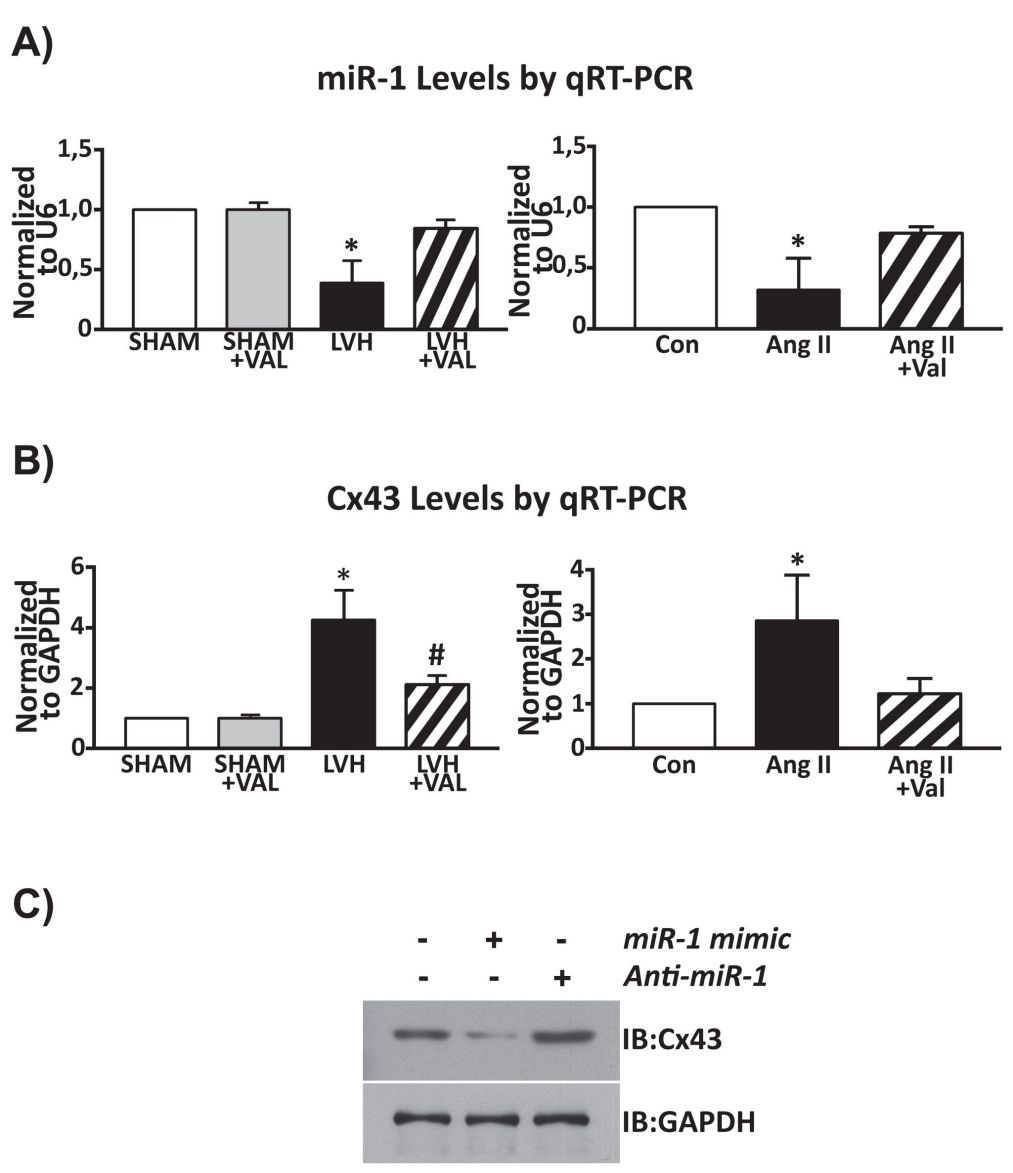
Molecular determinants of ventricular tachyarrhythmias in cardiac hypertrophy. Real time RT-PCR for miR-1 (A) and for Connexin 43 (B) levels. **A**: *left panel*, chronic pressure overload was associated with reduced miR-1 levels and increased transcript levels of Cx43 (showed in panel **B**, *left panel*) compared to SHAM and SHAM+VAL; in contrast, valsartan administration for twelve weeks after banding determined a restoration of miR-1 levels and reduced Cx43 levels almost to basal values (*p<0.05 vs. all, ^#^ p<0.05 vs. SHAM and SHAM+VAL). *Right panel*: cardiomyocytes stimulated with Angiotensin II showed lower expression levels of miR-1 compared to unstimulated cardiomyocytes (Con), with increased miR-1 levels in AngII+Val group (*p<0.03 vs. all, N=6); **B**, *right panel*: conversely, AngII challenge was associated with increased expression levels of Cx43, whereas valsartan reverted this phenomenon (*p<0.05 vs. all, N=6). **C**: cultured cardiomyocytes provided additional evidences, through gain- and loss-of-function assays, that Cx43 is a direct target of miR-1; cardiomyocytes were grown on 6-well plates to 70% confluence. Specific miR-1 mimic (30nM per well), and Anti-miR-1 (60nM per well) were transfected using siPORT NeoFX Transfection Agent (Ambion) according to the manufacturer’s protocol.

MiR-1 is abundantly expressed in skeletal and cardiac muscle, and it directly targets Cx43 for repression [34,35]. To investigate the relationship between electrical remodeling of pressure-overloaded hypertrophic hearts and miR-1 levels, we first assessed the differential expression of this miRNA in SHAM, SHAM+VAL, LVH and LVH+VAL rats. MiR-1 was significantly down-regulated in hypertrophic and arrhythmic group of rats (LVH) in contrast to sham-operated rats (-61% decrease, p<0.01 vs. all, Figure 7A, *left panel*). In hypertrophic rats treated with VAL, the expression levels of miR-1 were increased (115%) returning to the normal baseline values of the sham-operated groups (-16% decrease, p=NS). Furthermore, AngII stimulation significantly down-regulated (-68% decrease) miR-1 levels in cultured myocytes vs. un-stimulated cells (p<0.03 vs. all, N=6), whereas Valsartan administration reduced miR-1 down-regulation by AngII treatment *in vitro* (Figure 7A, *right panel*).

As above reported, Cx43 protein expression and phosphorylation is increased in hypertrophic cardiomyocytes of rats with aortic banding. Accordingly, we observed that Cx43 mRNA levels are also up-regulated in the LVH group (Figure 7B, *left panel*). Additionally, *in vitro* experiments showed an almost three-fold increase of Cx43 mRNA levels by AngII when compared to untreated cardiomyocytes (p<0.05, vs. all, N=6). Valsartan significantly reduced AngII stimulated Cx43 mRNA up-regulation in *in vitro* cardiomyocytes (Figure 7B, *right panel*). Thus, Cx43 mRNA and protein levels are up-regulated with a concurrent down-regulation of miR-1 levels in aortic banded hearts *in vivo* as it is expected by miR-1 direct targeting of Cx43 [32]. Indeed, we confirmed through gain (using a miR-1 mimic) and loss (Anti-miR-1) of function *in vitro* experiments that miR-1 directly modulates Cx43 levels (Figure 7C).

**Figure 8:**
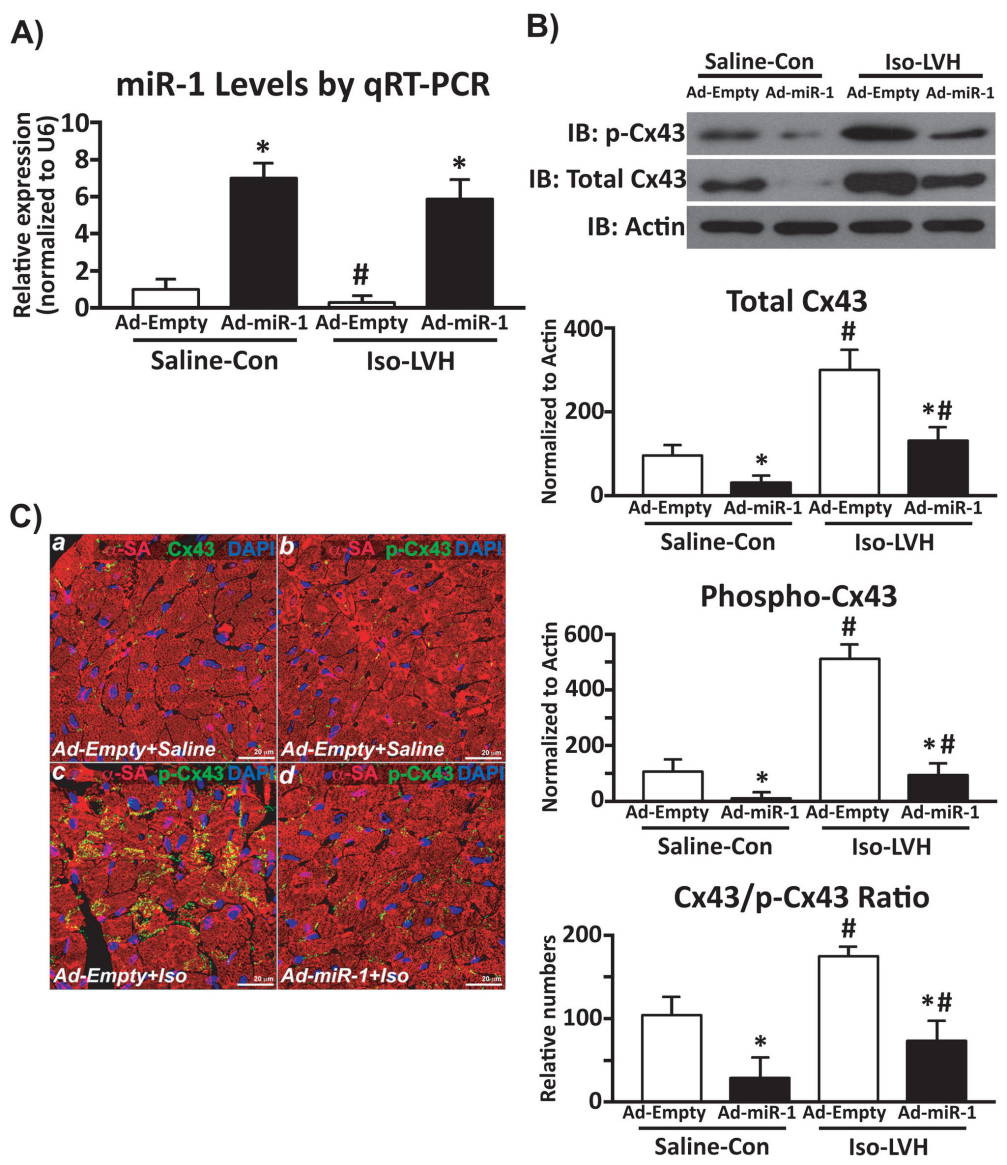
miR-1 modulates Cx43 activity in cardiac hypertrophy. **A**: cardiac levels of miR-1 after adenovirus-mediated cardiac overexpression in control saline-injected (Saline-Con) and in Iso-induced hypertrophic mice (Iso-LVH, *p<0.05 vs. Ad-Empty; #p<0.05 vs. Saline-Con). **B**: total and phosphorylated (Ser279/Ser282) Cx43 levels in normal and hypertrophic mouse hearts after Adeno-Empty or Adeno-miR-1 myocardial release (*p<0.05 vs. Ad-Empty; #p<0.05 vs. Saline-Con). **C**: immunohistochemistry and confocal microscopy in murine normal and hypertrophic heart sections; panels a-b show normal Cx43 expression in the gap junction (a) and very low level of its phosphorylation at Ser279/Ser282; ISO-induced LVH determined hyper-phosphorylation of Cx43 and its displacement from the gap junction to the cytoplasm of hypertrophic cardiomyocytes (c); panel d shows a significant reduction of phospho-Cx43 and its stabilization within the gap junction by adenovirus-mediated miR-1 selective intra-myocardial overexpression (*red*: α-sarcomeric actin, α-SA; *green*: Cx43 or p-Cx43; *blue*: DAPI).

Finally, we tested whether miR-1 plays a direct role in the modulation of Cx43 activity in an *in vivo* hypertrophic heart. To this aim, either an Ad-miR-1 or an Ad-Empty intra-myocardial deliver was performed in C57BL/6 mice and 72 hrs later the adenoviral-infected mice were treated with Isoproterenol (Iso) to induce LVH or just saline as control (Con) for 14 days. At sacrifice, miR-1 was correctly over-expressed in Ad-miR-1 treated mice when compared to Ad-Empty (Figure 8A). Iso induced significant reactive cardiomyocyte hypertrophy (cross sectional area, 306±18µm^2^ vs. 222±11µm^2^ in Con; p<0.05) and miR-1 down-regulation in Ad-Empty mice (Figure 8A). On the other hand, Ad-miR-1 overexpression prevented cardiomyocyte hypertrophic response to Iso treatment (cross sectional area, 233±12µm^2^; p=NS vs. Con and p<0.05 vs. Iso). Importantly, in agreement with the data on rat LV hypertrophy by aortic banding, miR-1 down-regulation by Iso-mediated hypertrophy was associated with Cx43 increased protein levels and enhanced phosphorylation (Figure 8B). The increased phosphorylation of Cx43 correlated with its displacement from the gap junction (Figure 8C). Importantly, miR-1 overexpression significantly reduced Cx43 protein levels with a concomitant significant reduction of its phosphorylated levels (Figure 8B). Accordingly, the gap-junction displaced and myocyte cytoplasmic accumulated hyper-phosphorylated Cx43 in hypertrophic hearts was significantly reduced in the Ad-miR-1 treated mice (Figure 8C). Thus, these data above provide the first direct evidence that miR-1 plays a major role in the modulation of Cx43 activity and location in *in vivo* cardiac hypertrophy.

Overall these data indicate that a hypertrophic stimulus on cardiomyocytes induces miR-1 down-regulation increasing the expression of Cx43, which in turn is phosphorylated by the hypertrophic stress-induced MAP kinases and so drifted away from the gap junction. The latter phenomenon leads to the increased susceptibility to develop life-threatening VT in the hypertrophic hearts. Intriguingly, an anti-hypertrophic agent, such as the AT1R blocker Valsartan, appears to exert its beneficial effects at least in part by attenuating this detrimental molecular pathway activation.

## Discussion

The main findings of this study document that: i) connexin 43 expression and activity (with its consequent displacement from the gap junction) increases in response to hypertrophic stress in cardiomyocytes *in vitro* and *in vivo*; ii) miR-1 directly targets for Cx43 repression and it is concurrently down-regulated in hypertrophic cardiomyocytes *in vitro* and *in vivo*; iii) molecular myocyte remodeling in cardiac hypertrophy increases MAPK-ERK1/2 activation, which in turn hyper-phosphorylated Cx43 and this shift redistributes Cx43 away from the intercalated disks favoring gap junction disassembling; iv) the hypertrophic myocardium is therefore prone to ventricular tachyarrhythmia (VT); v) angiotensin II type 1 receptor (AT1R) blockade reduces the maladaptive hypertrophic signaling inhibiting ERK1/2 activation while maintaining the pro-survival Akt function, attenuating miR-1 down-regulation and Cx43 displacement from the gap junction. The latter appears to be one of the molecular mechanisms modulated by AT1R blockade to reduce the life-threatening hypertrophic-dependent VT.

Cardiac hypertrophy is an independent risk factor for the development of arrhythmias, diastolic dysfunction, congestive heart failure and death, as assessed in experimental models and in humans [3,35–38]. The occurrence of malignant VT is determined by increased automaticity, enhanced heterogeneity of conduction, neuro-humoral changes, structural remodeling and alterations in expression and distribution of gap-junctions and ion channels [39,40]. In the mammalian heart, a connexin composed of 342 amino acid residues with a molecular weight of 43000 (Cx43) is the most abundant connexin. Hypertrophied rats have changes in quantity and subcellular localization of Cx43 [41]. In particular, the cell-to-cell electrical uncoupling of cardiomyocytes plays an important role in determining ventricular arrhythmogenesis during hypertrophy [5,37,42]. In this study we have shown that cardiac hypertrophy is characterized by an aberrant increased expression of Cx43, whose increased levels have been already reported in different experimental models in association with an abnormal subcellular localization. In fact, Cx43 is located in physiological conditions at the intercalated disks and lateralizes aberrantly toward the Z lines, or it is internalized in endosomes inside the cytoplasm in different pathologic conditions. Phosphorylation of Cx43 has been implicated in the regulation of gap junction communication at several stages of the connexin 'life cycle', including hemichannel oligomerization, export of the protein to the plasma membrane, hemichannel activity, gap junction assembly, gap junction channel gating and connexin degradation [32]. Cx43 phosphorylation is dynamic and changes in response to activation of many different kinases, including PKA (protein kinase A), PKC (protein kinase C), p34cdc2 (p34cdc2/cyclin B kinase), protein kinase CK1, MAPK (mitogen-activated protein kinase) and Src (pp60Src kinase) [33]. Treatment of cells with different growth factors is accompanied by increased Cx43 phosphorylation of serine residues with the net effect typically being a reduction in gap junction communication [43,44]. In particular, extracellular mitogen stimulation can also lead to increased phosphorylation of Ser255 and Ser279/Ser282, sites known to be MAPK family substrates [43]. Phosphorylation of Ser279/Ser282 is important in curtailment of gap junction communication, as these events are able to decrease gap junction channel ‘open time’ [44]. Additionally, the Src tyrosine kinase, an upstream regulator of MAPK activity, can also phosphorylate Cx43, and it negatively affects Cx43 activity by multiple mechanisms [45]. The renin-angiotensin system has been classically shown to be a major regulator of pathologic cardiac hypertrophy [6]. Indeed, Angiotensin II (AngII) is rapidly released from myocardial cells in response to cardiac damage and stretch; after binding to the AT1R, through Src, Ras, Gq and PKC mediation, it activates the maladaptive extracellular signal-regulated protein kinase 1/2 (ERK1/2) generating pathological cellular growth [20,30,46–49]. Valsartan, a classic non-competitive antagonist of the AT1R, has been widely used in the clinical setting for the treatment of patients with systemic hypertension and concentric cardiac hypertrophy [50]. However, its role in preventing VT that predispose to the risk of sudden cardiac death still remains to be elucidated. In the present study, valsartan administration significantly reduced the magnitude of cardiac hypertrophy and the incidence of arrhythmias, both as isolated ventricular ectopic beats in basal conditions and in the form of organized VT elicited after invasive provocative study, in rats after 12 weeks of pressure overload. In particular, AT1R blockade was able to reduce both ERK1/2 phosphorylation and ensuing Cx43 phosphorylation in vitro and in vivo, resulting in normalization of gap junction communications. Thus, these data lend support to a phenomenon whereby pressure overload-dependent release of mitogen stimuli like AngII activates ERK 1/2 that phosphorylates Cx43 reducing gap junction communication. This detrimental signaling mediates pathologic cardiac growth with the consequence of increasing malignant arrhythmias development.

Cx43 has been already established as a target of miR-1 [34]. The latter is dramatically reduced in cardiac hypertrophy, and contributes significantly to the overexpression of Cx43, hence determining part of the arrhythmogenic substrate of cardiac hypertrophy, whereas other receptors might be involved in maintaining other abnormal electrophysiological substrates [40]. Moreover, cardiac miR-1 levels have been found reduced in pathological conditions such as acromegaly [51]. Here we confirm these data and provide the first evidence that AT1R blockade prevents miR-1 down-regulation in hypertrophic stressed cardiomyocytes. Intriguingly, MAPKs are known regulators of miR-1/miR-133 biogenesis [52] and we have recently shown in vascular smooth muscle cell that ERK1/2 activation suppresses miR-133 expression [13], the miR-1 cognate bicistronic gene. Thus, it is tempting to speculate that ERK1/2 dependent signaling stimulated by cardiac stretch and AngII release mediates miR-1 down-regulation and Cx43 increased expression. The latter, as shown in the present study, is then hyper-phosphorylated by ERK1/2, decreasing gap junction communications, which may explain the increased risk of malignant VT in cardiac hypertrophy. Furthermore, miR-1 overexpression inhibits MAPK-ERK1/2 phosphorylation in hypertrophic cardiomyocytes *in vivo* [53]. The latter molecular adaptation provides a potential explanation of our findings showing that miR-1 regulates not only Cx43 expression through the expected gene silencing mechanism but it also indirectly modulates Cx43 activity. Indeed, we speculate that miR-1 overexpression leads to inhibition of ERK 1/2 phosphorylation *in vivo*, and the latter in turn prevents Cx43 phosphorylation and displacement from the gap junction. Nevertheless, further studies are still needed to prove this hypothesis.

Finally, in the present study we show that Valsartan-dependent reduction of cardiac hypertrophy induced by aortic constriction is associated with increased activated Akt levels (with a parallel reduction in ERK1/2 phosphorylation) when compared to rats with the sole aortic banding. While the latter is surprising considering the known role of Akt in cardiomyocyte growth [54], this finding generates the hypothesis that Akt activation in absence of concurrent ERK1/2 activation leads to an attenuated and beneficial adaptive cardiac response to pressure overload that improves cardiac function. Incidentally, Akt activation is indeed responsible for physiological cardiac growth by exercise training that is followed by an improved performance [55]. This beneficial adaptive activation of Akt in physiological hypertrophy as opposed to the detrimental signaling pathways (including the MAPKs) underlying pathological cardiac growth is the basis for these two different types of cardiac hypertrophy. It is also intriguing to speculate that Akt activation in absence of MAPK activity triggers a pro-endothelial secretory response in myocytes that increases capillarization during cardiac growth [56]. Nevertheless, it remains unanswered whether Akt has a role in modulating VT.

In conclusion, this study provides in vivo and in vitro evidences that the selective AT1R inhibition reduces total and phosphorylated levels of Cx43 through miR-1 expression normalization and ERK1/2 inhibition in hypertrophic stressed cardiomyocytes. Accordingly, the development of LVH, occurrence of hyperkinetic VT and reduction in cardiac function after pressure overload in an experimental rat model of ascending aortic banding are prevented by AT1R which is associated with the attenuation of miR-1 down-regulation and the consequent stabilization of Cx43 activity within the gap junction. These data increase knowledge on the signaling pathways responsible for LVH and the related mechanisms of VT, opening new avenues to the modulation of microRNAs as potential therapeutic targets in this clinical setting.
